# Supplementary material for: Transcriptional changes detected in fecal RNA from neonatal dairy calves of different breeds following gastrointestinal disease of varying severity
Source: PLoS One. 2022 Dec 1;17(12):e0278664. doi: 10.1371/journal.pone.0278664 (PMC9714867; doi:10.1371/journal.pone.0278664)
Supplement: Attachment — Submitted filename: Response to Reviewers.docx [file pone.0278664.s003.docx]

Thank you for your insightful questions and comments. Your commentary has led to changes that we believe make the manuscript a stronger addition to the literature. Please find responses to specific questions below.

Journal Requirements:

When submitting your revision, we need you to address these additional requirements.

1. Please ensure that your manuscript meets PLOS ONE's style requirements, including those for file naming. The PLOS ONE style templates can be found at

[https://journals.plos.org/plosone/s/file?id=wjVg/PLOSOne_formatting_sample_main_body.pdf](https://urldefense.com/v3/__https:/journals.plos.org/plosone/s/file?id=wjVg*PLOSOne_formatting_sample_main_body.pdf__;Lw!!JmPEgBY0HMszNaDT!qk8TQ0H220O3b0crkzVsbIAF34q4Se_ymFGCdNDtqqr3x0PG4YV-m7H2eLkgHTNF1hRAlQAimUIMNZCH$) and

[https://journals.plos.org/plosone/s/file?id=ba62/PLOSOne_formatting_sample_title_authors_affiliations.pdf](https://urldefense.com/v3/__https:/journals.plos.org/plosone/s/file?id=ba62*PLOSOne_formatting_sample_title_authors_affiliations.pdf__;Lw!!JmPEgBY0HMszNaDT!qk8TQ0H220O3b0crkzVsbIAF34q4Se_ymFGCdNDtqqr3x0PG4YV-m7H2eLkgHTNF1hRAlQAimbBv9BbN$)

2. Thank you for stating in your Funding Statement:

“This project was supported by the WSU CVM Caldwell Endowment (CSM), the American Jersey Cattle Association (CSM), and the USDA National Institute of Food and Agriculture’s Animal Health & Disease Research Capacity Grant project 1014680, and Agriculture and Food Research Initiative Competitive Grant no. 2019-68008-29897 (CSM). The funders had no role in study design, data collection and analysis, decision to publish, or preparation of the manuscript.”

Please provide an amended statement that declares *all* the funding or sources of support (whether external or internal to your organization) received during this study, as detailed online in our guide for authors at [http://journals.plos.org/plosone/s/submit-now.](https://urldefense.com/v3/__http:/journals.plos.org/plosone/s/submit-now.__;!!JmPEgBY0HMszNaDT!qk8TQ0H220O3b0crkzVsbIAF34q4Se_ymFGCdNDtqqr3x0PG4YV-m7H2eLkgHTNF1hRAlQAimYJJlVGe$)  Please also include the statement “There was no additional external funding received for this study.” in your updated Funding Statement.

Please include your amended Funding Statement within your cover letter. We will change the online submission form on your behalf.

3. Please include captions for your Supporting Information files at the end of your manuscript, and update any in-text citations to match accordingly. Please see our Supporting Information guidelines for more information: [http://journals.plos.org/plosone/s/supporting-information](https://urldefense.com/v3/__http:/journals.plos.org/plosone/s/supporting-information__;!!JmPEgBY0HMszNaDT!qk8TQ0H220O3b0crkzVsbIAF34q4Se_ymFGCdNDtqqr3x0PG4YV-m7H2eLkgHTNF1hRAlQAimWoDST4u$).

**AU:** Thank you for pointing out our editorial mistakes. We have corrected various file names, amended the Funding Statement and included it within the Cover Letter, and updated the caption for the Supporting Information.

Review 1 Queries:

The article titled “Transcriptional changes detected in fecal RNA from neonatal dairy calves of different breeds following gastrointestinal disease of varying severity” by C.S. McConnel and collaborators explores an important topic in the dairy industry, focusing the changes in the transcriptome of preweaning calves in response to mild or clinical GI events. The comparison between breeds, as well as the strategy chosen to quantify gene expression, are novel and interesting. However, the manuscript presents several areas that must be improved before it can be deemed suitable for publication. I will list a few broad observations, followed by more precise corrections.

1) Presentation of the experimental design, and statistical soundness: the way the experimental design is explained is confusing. The cohort was housed on a single facility, to which the animals were transported; later, the authors mention that the calves were sourced from different farms (I can’t tell if each breed came from a separate farm, or if every farm provided equal numbers of calves of both breeds). Because of the abstract, I assume all calves were heifers, but the authors do not mention it in the methods. It’s unclear if calf weight and daily grain intake (when offered) were measured, and it appears that the milk intake was only assessed qualitatively. I’m also still unsure as to when the fecal samples were collected: I understand that calves were 4 to 12 days old at collection #1, then were recollected for DEG 10 days later. Except if they were already showing signs of scours, then they were sampled whenever they first presented symptoms + 10 days later? Another reading is: all were sampled initially, then again if they presented scours before d +10, and then twice more (at collection#1 + 10 days and at first symptoms + 10 days). This is very confusing, especially since I can’t understand which sample was ultimately used for DGE in the scours or systemic group, and I have a suspicion that healthy vs non-healthy samples were collected at different time points.

**AU:** Thank you for identifying points in need of clarification. Calf weights and daily grain intakes were not measured due to logistics and housing constraints on the ranch. Milk intake was assessed qualitatively as stated ((good appetite; did not finish the milk or did not consume any of what was offered; orogastric intubation). We have added clarifying text as follows:

1. Breed differences were variable within the source dairies ranging from Holstein and Jersey, Holstein or Jersey only, or crossbred from a single source..
2. Heifer calves were randomly enrolled.
3. The section “Fecal samples for differential gene expression (DGE)” and Fig 1 both have been edited in an attempt to more clearly define the following:
   a. Days of age 4-12
      i. Period of age during which calves were randomly enrolled and an initial (day 1) fecal sample was collected for both a fecal consistency score and microbiome analysis.
      ii. Calves that were initially sampled when healthy or with scours but progressed to systemic enteritis (i.e., diarrhea plus a behavioral score ≥1) during this period were sampled again on the day that systemic enteritis was diagnosed.
      iii. Calves with systemic enteritis prior to a random sample were sampled once during this period at the time of initial clinical diagnosis irrespective of the randomized sampling scheme.
   b. Days of age 13-21
      i. Period of age during which follow-up (day 10) fecal samples were collected for both a fecal consistency score and DGE analysis.
      ii. Calves that were initially sampled when healthy or with scours but progressed to systemic enteritis by 12 days of age were sampled twice during this period—9 days following both the first (healthy or scours) and second (systemic enteritis) samples to provide 2 day 10 samples.

A key point here is that calves that were randomly enrolled were identified as having scours (or not) when initially sampled whereas systemic enteritis was diagnosed based on clinical presentation. Calves with systemic signs were sampled at the time of diagnosis during the 4-12 days of age window regardless of whether they had already been sampled based on their random allocation. Ultimately one of sample was analyzed for DGE with preference given to the systemic enteritis sample. Contrary to suspicions, healthy and non-healthy samples were collected at the same time.

If we account for disease status and time, there should be 5 groups: healthy throughout, healthy to scours, scours to healthy, systemic to healthy and healthy to systemic; other groups, (systemic to scours, scours throughout, systemic throughout) are absent or have too few samples (and they often received different treatment). All of this separated by breed, so 10 groups for pairwise analysis. However, in the presentation of the results, several contrasts are presented, some of which I do not fully understand: what is the comparison in table 6? Is it each non-healthy group vs healthy at DEG? Healthy throughout? In table 8: the comparisons are based on the time of systemic enteritis and are compared to “healthy calves of the same breed”. I assume “prior” means systemic to healthy ; does that mean that they’re compared to all calves that stated out as healthy, regardless of whether they were healthy throughout, or healthy to scours? Or does it imply healthy throughout?

**AU:** Indeed there are a number of possible groups—including systemic to scours and scours throughout which are demonstrated in Figs 2 and 3 (no calves survived to populate a group of systemic throughout). To help alleviate confusion, the Table 6 legend has been moved into the title to explain that Table 6 is presenting log fold-changes for fecal mRNA gene expression in calves with scours or systemic enteritis at any point from enrollment until the day 10 DGE sample as compared to healthy calves of the same breed(s).

Thank you for raising the question regarding Table 8, as it highlighted that we should add “consistently” to the descriptor for healthy calves within the titles of Tables 5-8 (i.e., healthy to healthy category), and additional text to Table 6 clarifying categories for comparison (e.g., scours to healthy and healthy to scours). Within the title for Table 8 specifically, we have also included text clarifying that calves with systemic enteritis ≥7 days prior to the day 10 DGE fecal sample were within the “systemic to healthy” groups, whereas calves with systemic enteritis on the day of the DGE fecal sample were within the “healthy to systemic” category.

We discussed how best to present this data given that less useful information was garnered from the collapsed data (i.e., comparing calves with clinical symptoms of GI disease at any point from enrollment until the day 10 DGE) than from the more nuanced analyses of disease severity culminating in the findings presented in Table 8 for systemic disease. In the end, we decided it was important to explore the data in a stepwise fashion in an effort to peel back the layers from broad disease and combined breeds to refined disease and breed-specific outcomes. Although in the grand scheme of things those outer "layers" of the analyses were overly generalized given the final specific outcomes, we felt that omitting those general assessments would lead readers to overlook the importance of the disease classifications. As we stated in the Discussion, these findings highlight the importance of discriminating between breed-specific GI disease phenotypes when describing, comparing, and investigating the overall consequences of disease on calf health and wellbeing. In other words, we are hoping that this approach highlights the fact that scours (diarrhea) should not be considered a singular disease state without consideration for other contextual aspects such as breed and severity.

Finally, the groups are also different in size, and I’m not sure if the authors made any adjustments to their statistical approach to account for unequal group sizes. As it stands, a better explanation of sample collection schedule, group assignment and statistical analysis is required. I suggest analyzing based on a combination of disease progression and breed, leading to the aforementioned 10 groups, though I’m not certain that the statistical power will be maintained.

**AU:** We truly appreciate your overall insights and identification of issues with clarity. We trust that our edited Fig 1 timeline and additional text throughout has provided the necessary clarity to more easily follow the sampling and analytic sequence. As for adjusting our statistical approach to account for unequal group sizes, nSolver Advanced Analysis runs the R package MASS which accounts for unequal variance when estimating parameters and their variance by estimating a dispersion parameter (𝜙) within the negative binomial model. It accommodates the variance of probe expression within biological replicates, which is not of interest in differential expression analysis. When 𝜙 = 0, the negative binomial model reduces to the Poisson model. It is worth noting that the unequal group sizes were a function of both on-farm disease incidence and the mandatory exclusion of 99 samples (n = 38 consistently healthy; n = 47 scours; n = 14 systemic enteritis) due to insufficient fecal mRNA raw counts or expression above background. It was unfortunate that we were unable to use that data, but the reality is that fecal RNA extraction is challenging, and we were glad to have as many useful samples as we did even it did mean that we could not look more closely at select groups lacking a baseline number of samples (i.e., scours to scours, n=2; systemic to scours, n=2). In the end, although we had unequal numbers of animals per group (healthy, scours, systemic disease) and by breed (Jersey, Holstein) we were satisfied that the various comparisons met baseline criteria for sample size and the inclusion of all available data was more useful than restricting data in the interest of equal group sizes.

2) Presentation of results: tables are great for a detailed presentation, but virtually impossible to use for cross-group comparisons. I strongly suggest the table be added to the supplementary files, and be replaced with a grouped column graph: the x axis containing the name of the gene of interest, a group of 3 columns per gene (Holstein, jersey, combined), one separate panel per contrast, and some kind of symbol to indicate significance. I’m partial to fold change, rather than log2FC, but both are acceptable; although, please ensure that whatever is chosen is referenced consistently throughout the text (see 242, 311, 326, 333 and other points where it's called “log fold change”). The heat maps are fine, but a per-sample view does easily display group means and is not terribly useful in this case. I suggest to move that to supplementary as well.

**AU:** We have edited log fold-change to log_2_ fold-change where necessary.

Admittedly, the Tables as presented provide a lot of information through which to sift. However, we respectfully disagree that they should be replaced or added to supplementary files. As we stated above, we think it is important to discriminate between breed-specific GI disease phenotypes and we think that these Tables provide a worthwhile mechanism for doing so. Although simplified versions in the form of columnar graphs might be more visually appealing, they would almost certainly limit our ability to display the full range of data including standard and corrected p-values, which we believe are important for exploring “significant” differences as well as plausible trends in the data. Our goal is to be as transparent as possible with regard to the findings from this study, and we think that presenting the data as-is per comparison is the best (albeit dense) mechanism for doing so. We have proceeded in this manner previously (<https://doi.org/10.3389/fvets.2020.559279>), and the second Reviewer for this manuscript appears to agree given their comment that this manuscript is easy to understand.

We will certainly defer to the Editor, but we would also respectfully disagree with moving the heatmaps to the supplementary files. Our experience discussing this data with veterinarians has been that the heat maps provide one of the more powerful representations of the data as it pertains to both individual variations and groups. As such, we would prefer leaving these Figures within the main body of the text as a visual prompt similar to what might be achieved through the addition of columnar graphs.

3) Reference to microbiome study: the authors refer to a 2022 microbiome study, also published in PLOS, which as I understand was performed on the same samples/animals. The authors should try to combine these findings with those and see if there are any links.

**AU:** The previously published study was a bit more expansive and involved additional samples taken from throughout the summer of 2019. We considered exploring the overlaps between microbiome and DGE findings but have shelved that idea for the time being due to time and monetary constraints.

Other notes (with line number)

57: the statement about how information is “wasted” is subjective. Please rephrase

**AU:** This has been changed to “unproductive”.

60-61: I’d argue that the welfare of animals is a more relevant implication than the reputation of the dairy industry…

**AU:** Agreed. That is why we say that these misspecifications of disease processes can result in inappropriate therapeutic and preventive actions that impact lifetime wellbeing. Nonetheless, the reputation of the dairy industry is at risk as well and that is an important consideration.

78-79: depending on how the statistical approach is modified, it may not be worth mentioning the therapeutic approach, since it was not treated as a source of variation.

**AU:** Although not a major component of the analysis, we think that therapeutic interventions should be mentioned simply because they were a component of the management of systemic enteritis.

90-95: please specify number of calves enrolled, how many came from which farm, whether they were all female or not.

**AU:** These data now are specified within the first paragraph of the Results including reference to the fact that all enrolled calves were female.

120: how much grain was offered at the start?

**AU:** There was no set amount initially—it was literally a handful. That has been clarified within the text.

127: missing comma between Tulare and CA

**AU:** Corrected.

139: can the raw data for rectal temperatures be provided in the supplementary?

**AU:** We decided to only include rectal temperatures in Table 4 to highlight the variability in rectal temperatures even in those calves with systemic evidence of disease. Our experience with rectal temperatures on dairies/calf ranches in the western US in general and with this project specifically is that they are highly variable and ultimately of limited value in clarifying GI disease severity in calves with scours.

147 and elsewhere: please add catalog number for solutions/reagents used

**AU:** Solutions such a lactated Ringer’s can be sourced from multiple outlets as can be other of the products used on farm or in the lab. Unless we have overlooked a new PLOS ONE Best Practices in Research Reporting we think that the guidelines mandate that company name and location are the standard practice for reporting solutions and reagents that have a specific origin.

182: homogenized how?

**AU:** To avoid any confusion here we restated to say that the samples were thoroughly mixed by vortex.

187: “was” should be “were”

**AU:** Corrected.

188: why the PBS? Also, homogenized how? 189-192: I don’t understand the strategy; first they were vortexed and the solids were removed, then the supernatant was centrifuged again and discarded? Why was this done instead of bead-homogenization and removal of solids, then lysis?

**AU:** The PBS and strategy was based on a recommendation from Fisher Scientific’s technical help; we wanted host material rather than everything from the sample. Text has been added to explain and to restate the homogenization.

196: report average yield, 260/280 and 260/230 ratios. 198: report mean RIN or equivalent

**AU:** Due to the instability of fecal RNA and Nanostring’s allowance for DV values as a quality measurement, RNA integrity values were not used for quality assessments. Instead, samples with DV200 (percentage of RNA fragments >200 nucleotides) values >50% and concentrations >20 ng/uL were submitted for diagnostic processing at the Primate Diagnostic Services Laboratory.

202: how was this threshold selected?

**AU:** Great question. This was based on discussions with Nanostring’s Field Application Scientist, Dr. Lisa Poole. The short answer is that the Nanostring manual suggests the use of DV200. The longer answer is that there are published instances in the literature that show RIN is not indicative of how successful a gene expression experiment with nCounter will be due to the fact their chemistry does not rely on total RNA integrity that is measured by RIN. Instead, since the barcodes only need about 100bp of intact RNA to bind, it only matters that the RNA is at least 200bps in length, hence the dependence on the DV200 value over RIN.

205-208: were the re-extracted samples quantified for DV200 before submission?

**AU:** Re-extracted sample DValues were not quantified. Trizol extracts that were equal to or greater then 35ng/ul were allowed to move forward in the process and all re-extracted samples were well above 35ng/ul. In fact, only re-extracted samples with concentrations >60ng/uL were submitted for diagnostic processing per Nanostring recommendations.

210: I’m unfamiliar with the system, but is it possible to get more detail on probe design?

**AU:** This is probably more than you care to know but the probe design process used by Nanostring breaks a target transcript down into 100nt windows to profile for probe characteristics.  Each 100nt window is profiled for intrinsic sequence makeup – non-canonical bases, GC%, inverted and direct repeat regions, runs of polynucleotides, as well as calculating the Tm for each potential probe to target interaction.  Each individual probe is then thermodynamically tuned to determine the optimal probe length (each probe ranges in size from 35-50nt) within the 100nt target region.  Next, a cross-hybridization score is calculated for each probe region; BLAST is used to identify potential off-target interactions, and then an internal algorithm is used to determine the cross-hybridization score using a pair-wise comparison of the probes to the potential cross-hybridization target.  In addition to a cross-hyb score, a splice isoform coverage score is generated by identifying transcripts that are isoforms to the transcript the probe is being designed to.  Once all of this information is compiled, then the final probe is selected by identifying the probe that has the optimal splice form coverage, the best cross-hybridization score, and the most optimal thermodynamic profile.

To ensure that there are no potential intramolecular probe-probe interactions that could cause elevated false-positive or background signal for any individual probe in a CodeSet, a stringent intramolecular screen is run on every collection of probes assembled into a CodeSet.  This screen is run using a sensitive algorithm that calculates both the Tm and the free energy potential of every probe compared to every other probe in the project.  If a probe is identified having either Tm or free energy profile above a conservative threshold, an alternative probe is selected for that target and the screening is re-run until there are no issues.

We considered including some more of this information in the Materials and Methods, but it seems overkill under the circumstances and we felt the inclusion of a relevant reference was more in line with expectations (<https://doi.org/10.1186/1756-0500-2-80>).

263: I’m not sure this whole table needs to be here. Maybe a per-group summary of TSP and ages, and then this table could go in supplementary files.

**AU:** This is a bit of a toss-up between providing excessive background information and doing our best to help set the stage for the comparisons to follow. We would prefer leaving this Table as-is even if it is a bit of overkill, to help readers appreciate the enrollment/sampling scheme that is presented in Fig1 while highlighting the various treatments of systemically affected animals.

333 and other tables: check consistency on tables. E.g. for table 7, the timing of disease is indicated as day 1 or day 10, while for systemic it’s prior and current. I know they’re not exactly the same in terms of time, but it’s very confusing.

**AU:** Thank you again for pointing out this issue with clarity. We think it has been addressed appropriately per the edits discussed above.

377 and elsewhere: you can use “p-adj” or “adjusted p” instead of “B-Y P”

**AU:** This is true but we think that B-Y P helps the reader maintain a consistent understanding of where the adjusted p-value originated per the description in the Materials and Methods.

379 and elsewhere: the fold change here is missing the unit

**AU:** As mentioned above, we have edited log fold-change to log_2_ fold-change where necessary and included text in the Differential Gene Expression section of the Results indicating that Log_2_ fold-changes are ratios.

469-418: the discrepancy between the presented results and the Rosa et al. is not completely addressed. Add a sentence after 481 that ties it together

**AU:** Thank you very much for highlighting this section. The findings from that paper by Rosa et al. were not well represented in this portion of our manuscript. We should have been pointed out that there was a time-dependent *IFNG* downregulation in the Jersey calves following mild diarrhea; however, Rosa showed a pattern of fecal score and pro-inflammatory blood biomarkers (peaking both between 2 and 3 weeks of age) that indicated a pro-inflammatory response during diarrhea in neonatal dairy calves. This has been corrected within the text and provides a more egalitarian representation of the comparative data.

494: is there evidence in the literature of PMN transmigration in response to scours/diarrhea?

**AU:** This is a good question. There appears to be little in the way of direct evidence although this paper (https://doi.org/10.1128/IAI.67.9.4950-4954.1999) does suggest that Salmonella-induced enteritis leads to an inflammatory response as evidenced by a rapid and large influx of polymorphonuclear leukocytes into the intestinal mucosa and lumen and a net fluid secretion into the intestinal lumen. Rosa also mentions a good paper by Brazil et al. that speaks to the fact that the migration of PMN across the intestinal epithelium is a common pathological event of many mucosal inflammatory diseases (Brazil et al., 2010). We now have included this reference in our manuscript.

Please cite 512 and elsewhere: note a recent follow-up paper by Rosa et al. on the same calves as [6] that looks at expression of the same genes throughout the GI tract using tissue samples <https://www.sciencedirect.com/science/article/pii/S0022030220309115>.

**AU:** Done.

520: does “not noteworthy” mean “not statistically significant”?

**AU:** More or less—this seemed like an efficient method for pointing out the consistent downregulation without getting mired in B-Y or standard p-value cutoffs.

535-554: I’m not sure I agree with the whole argument here. By the logic applied in ~494, KRT8 could indicate larger degree of PMN migration into the lumen, as Rosa and Osorio in ref. 12 noted that KRT8 was downregulated in PMN; at the same time, the study in ref. 25 demonstrates decreased leukocyte migration and chemotaxis. Additionally, could breed differences have been skewed by unequal sample sizes and the contrasts that were chosen? The differences between the two breeds seem minimal (no significant p-adj, only unadjusted p).

**AU:** These are good considerations and worthy of debate. The key from our standpoint is that although Rosa and Osario demonstrated that mRNA expression of *AQP3* tended to be greater in PMN than fecal RNA (indicating that the fecal RNA samples in our study may have contained PMN mRNA transcripts), the more important point is that Rosa et al. also demonstrated that even under nondiarrheic conditions RNA isolated from neonatal dairy calf feces is derived from a considerable number of IECs as evidenced by mRNA expression of *KRT8*. Based on this latter finding we feel comfortable positing that the evidence for *KRT8* downregulation in Jersey calves with GI disease may have indicated adversely affected intestinal barrier protection and inflammatory regulation.

As mentioned above, we are confident that nSolver (R package MASS) appropriately accounted for any variance difference and adjusted the estimated model variances for over-dispersed data. That said, we certainly hope that our research and others will continue to explore breed differences in response to GI disease states.

565: What markers of disease resilience? Any insight on resilience could be assessed by looking at animals that recovered (scours to healthy and systemic to healthy) vs those that didn’t (scours to scours and systemic to systemic, which are both absent).

**AU:** Additional calves representing ongoing disease processes such as systemic-systemic, scours-scours, or systemic-scours would have provided a nice comparative group by which to evaluate potential markers of animal’s resilience based on balancing pathogen elimination (resistance) and tissue damage (tolerance). Hopefully we or others can continue with this line of inquiry to understand varying responses to disease more fully. That said, even without those comparative groups the genes that were selected for analysis based on previous work by Rosa et al. provide insight into resilience based on both breed and disease severity as demonstrated through divergent susceptibilities and apparent immune function.

Review 2 Queries:

Congratulations on the manuscript. It is well written and easy to understand.

**AU:** Thank you. We appreciate your kind words.

I will list just a few comments:

L21-23 would be "observed by Rosa et al. in fecal..."?

**AU:** Corrected.

L91 how many animals were previously evaluated in your study? And what are the selection criteria for the animals that you collected fecal samples from?

**AU:** We trust that our edited Fig 1 timeline and additional text throughout have provided the necessary clarity to more easily follow the sampling and analytic sequence. Specifically, heifer calves were randomly enrolled into the sampling scheme and allocated to an initial sampling day (day 1 enrollment sample) between 4 to 12 days of age to have fecal samples collected. This provided the opportunity to sample healthy calves and calves with previously undiagnosed scours but no other clinical abnormalities. Calves that demonstrated systemic enteritis (i.e., diarrhea plus a behavioral score ≥1) during this same period but prior to a random sample were sampled once at the time of initial clinical diagnosis irrespective of the randomized sampling scheme. Nine days after the initial fecal collection each calf was sampled again (day 10 DGE sample; 13-21 days of age) in an effort to align the fecal RNA transcriptomic analysis with previously documented delays in gene expression following evidence of diarrhea. Calves that were initially sampled when healthy or with scours but progressed to systemic enteritis by 12 days of age, were sampled again on the day that systemic enteritis was diagnosed. These calves then were sampled twice more 9 days following both the first (healthy or scours) and second (systemic enteritis) samples to provide 2 day 10 samples. No calf was sampled more than 4 times in total and only one sample ultimately was analyzed for DGE, with preference given to the sample obtained during systemic enteritis. Ultimately, a total of 183 heifer calves were enrolled with RNA extracted from fecal samples and submitted for diagnostic processing.

L91-93 How much colostrum was offered? And what was the concentration of IgG?

**AU:** Calves were fed colostrum at the dairy of birth which unfortunately means that there were no records available for specific levels of colostrum that were fed to individual calves. However, transfer of passive immunity was assessed via blood samples obtained from 1-day old calves via jugular venipuncture to measure total serum protein (TSP) using a calibrated refractometer (details below).

L93-95 did the calves have adequate transfer of passive immunity? What is the average value of TSP? Did any calves have failure in transfer of passive immunity?

**AU:** Average TSP was 6.5 ±0.8 g/dL. The individual TSP levels are presented in Table 3 and interestingly the average TSP was the same for healthy (6.5 ±0.9 g/dL) and diseased calves (6.5 ±0.8 g/dL); however, as expected the average TSP for Jerseys (6.9 ±0.7 g/dL) was greater than for Holsteins 6.0 ±0.7 g/dL; P < 0.0001).

L113-114 what was the mixture ratio? What is the composition of the liquid diet?

**AU:** The milk blend consisted of pasteurized waste milk together with milk replacer and was targeted for an optimal composition of 13% solids, 22-24% fat, and 28% protein.

L118 what is the composition of the oral electrolyte solution?

**AU:** The oral electrolytes were a proprietary product for the participating calf ranch, which unfortunately meant that we were not privy to specific compositional details.

L120-121 what is the composition of the concentrate?

**AU:** Similarly, the concentrate was specifically developed for the calf ranch as a proprietary product so we were provided only with the fact that it consisted of pellets, molasses, and whole corn.

L129-140 what are the references used for such measurements?

**AU:** Thank you for this comment as it highlighted the need for an appropriate reference that was missing: McGuirk SM. Disease management of dairy calves and heifers. Vet Clin North Am Food Anim Pract. 2008;24(1):139-53. Epub 2008/02/27. doi: 10.1016/j.cvfa.2007.10.003. PubMed PMID: 18299036.
