# Supplementary material for: Transcriptional changes detected in fecal RNA from neonatal dairy calves of different breeds following gastrointestinal disease of varying severity
Source: PLoS One. 2022 Dec 1;17(12):e0278664. doi: 10.1371/journal.pone.0278664 (PMC9714867; doi:10.1371/journal.pone.0278664)
Supplement: Attachment — Submitted filename: Response to Reviewers.docx [file pone.0278664.s004.docx]

Thank you for your additional input. questions and comments. Please find responses to specific questions below.

Reviewer #1: I would like to commend the authors for making pertinent changes to the manuscript, which has improved as a consequence. I still have a few comments to share, which I will list below in no particular order. If line numbers are provided, they refer to the version with track changes.

1) Regarding presentation of timeline and sample collection: I think I now understand how samples were collected. Let me try to lay it out with an example, so that the authors can confirm if I’m correct in my interpretation:
- If a calf is healthy or has scours at enrollment date and DOES NOT develop systemic enteritis throughout the study, two samples are collected, 9 days apart. Age at collection of sample 1 is 4-12 days, therefore age at sample 2 is 13-21 days. Sample 2 is used for DGE.

**AU:** Correct.

- If a calf is healthy or has scours at enrollment date, and DOES develop systemic enteritis throughout the study, sample 1 is collected as above, another sample is collected when symptoms show (let’s call this Sample 1_SE), then sample 2 is collected 9 days after sample 1, and Sample 2_SE is collected 9 days after Sample 1_SE. Ultimately Sample 2_SE is used for DGE.

**AU:** Correct.

- If a calf started with systemic enteritis at enrollment date, Sample 1_SE is collected at beginning of symptom, Sample 1 is collected as above, Sample 2 and Sample 2_SE are collected 9 days after corresponding sample. (however, based on Table 3, no animal that started SE had SE at the time the DGE sample was collected).

**AU:** Calves with systemic enteritis prior to selection for a random sample were sampled *only once* during this period at the time of initial clinical diagnosis. I.e., there was no Sample 1 collected if a Sample 1_SE preceded the anticipated randomized sample. We have added the word ‘only’ to the text and Fig1 to help clarify this point.

You are correct that no surviving calves that started SE remained SE 9 days later.

If the explanation outlined above is correct (and do please correct me if I’m wrong), I have a few comments:
- The explanation of the collection timepoints can be more effectively outlined with something like a flow chart that looks at initial enrollment state, condition throughout the 10 days (healthy, scours, SE), and state at DGE sample. The actual days of age of the calf can be added on a vertical bar next to the graph. In my opinion, the figure that was added in the first round does little to clarify the timeline, as it’s still based on the written description provided with the figure.

**AU:** Figure 1 has been updated to include a flowchart indicating observed enrollment and DGE sample classifications.

- Does table 3 discriminate based on disease progression? For example: if a calf started healthy, quickly developed SE, and was then treated and recovered before the DGE sample, this would show as healthy-healthy, just like a calf that started healthy and ended healthy without change? Maybe there are no such samples? Or does “enrollment sample” in Table 3 refer to “early sample” which would be Sample 1 or Sample 1_SE (see above) depending on whether they developed systemic enteritis or not?

**AU:** You are correct that ‘enrollment sample’ refers to the sample preceding the DGE sample. E.g., for the purposes of our analysis it was the sample used to enroll a calf into the study. Only one sample ultimately was analyzed for DGE. E.g., no samples from a calf with systemic enteritis were used for anything other than evaluating healthy to systemic, systemic to healthy, or systemic to scours. This might not have been clear given our previous word choice stating that preference was given to the sample obtained *during* systemic enteritis. This has been corrected within the text to clarify that preference was given to the sample obtained *during or following* systemic enteritis.

- Since the sample used for DGE are +9 days from sample 1, but sample 1 was collected later for animals that developed systemic enteritis, would this mean that animals that developed systemic enteritis are always slightly older than healthy/scours animals at the DGE sample?

**AU:** Correct. Ages were slightly different. Per Table 3, calves that survived following systemic enteritis were initially identified between 8-12 days of age, leading to a DGE sample obtained between 17-21 (average 19.6) days of age. All other DGE samples, including those obtained from calves that were initially healthy but developed systemic enteritis by the time of their DGE sample, were between 13-21 (average 16.4) days of age. Prior to conducting this study, it was decided that the sampling timeframe for DGE between 13-21 days of age would be analyzed in bloc given the relatively narrow spectrum of development. Ongoing research breaking down transcriptomic and DGE changes by day of age may ultimately prove enlightening as we continue to try and understand developmental aspects underlying immunologic resilience (resistance and tolerance).

2) Regarding the catalog numbers: both the authors and myself will agree that for common reagents such as lactated Ringer’s, PBS, or certain types of cell culture media adding the catalogue number may be superfluous. However, several nucleic acid extraction and processing kits have been cited within the manuscript, for which many formats (with slightly different chemistries) exist. A few examples: MagMAX Total RNA comes as a 96-preps kit in deep well plates, or as mirVana (still under the “Total RNA” umbrella) which maintains small RNAs. There are four commercial versions of the Qubit fluorimeter available. Invitrogen’s “RNA Storage Solution” (line 205) could be “THE RNA Storage Solution” from Thermo Fisher, or it could be RNA Later which the authors cite multiple times. Whirl Pak bags come in several formats (filtered, unfiltered, in different sizes etc.). The RNA Clean & Concentrator Kit from zymo comes as spin columns, loaded plates or magnetic beads…

Having worked with fecal RNA multiple times in the past, I have first hand experience with how sample collection vessels, storage temperature and solution, and extraction and cleanup can affect quality and integrity of the resulting product. I think it is more than reasonable to request that the author add catalog numbers to these products. The PLOS guidelines are not specific on this, but do request that catalog number be used when antibodies or cell lines are cited in the manuscript, which I think is a rather similar situation to the one we’re discussing here. Please, add the catalog number.

**AU:** Done.

3) Regarding probe design: my question aimed at the possibility of obtaining actual sequences for the probes. If the method used to design them follows the manufacturer’s design and is openly available online, then I will agree that information on the design strategy does not need to be included; the probe sequence, however, would be desirable. One additional note: I could not identify, in the extensive explanation of the probe design provided by the authors, if the probes are designed to span two exons (that is, to straddle an exon-exon junction). If that is not the case, please specify if the TURBO DNAse step of MagMAX was executed or omitted.

**AU:** An additional Supplementary Table 1 (S1 Table) has been added to provide the actual sequences for the probes. Per Nanostring’s Field Application Scientist, **t**he probes are designed based on transcriptomics data with the introns spliced out. Some may fall across exon-exon junctions, although others may not for targeting the RNA from that gene. So, if the question is how do we know the probe is not targeting DNA, if it is not covering a exon-exon junction unique to RNA? Then the answer is that the probe hybridization assay takes place at 65°C, and the temperature in the PCR block never rises past this so all genomic DNA remains double stranded and inaccessible to the probes, only single-stranded RNA should be binding.

4) RNA integrity vs DV200: the requirement for RIN or equivalent in gene expression studies is a debated topic, especially for methods that rely on amplification of short sequences such as RT-qPCR. The crux of the matter is as the authors indicate, amplified sequences are rather short, and as such are less likely to be impacted by degradation. This is true of both qPCR and the nanostring technology employed here, provided the amplicons in qPCR are <200 in length (which they often are). Which is to say, the conversation about RIN requirements is as pertinent to this manuscript as it is to qPCR. The authors mentioned in the response to my feedback that there are “published instances” in the literature where RIN was not indicative of successful quantification. I invite the authors to include a statement about the use of RIN vs DV200 to quantify transcript integrity, citing said manuscripts as support for their strategy. Additionally, the fact that re-extracted samples were not quantified for DV values is a limitation of this study and should be stated or justified, as there can be no guarantee on the quality of those samples, regardless of the method to quantify said quality.

**AU:** I think it is important to note that the NanoString technology does not used any “amplified” sequences. There are no enzymatic steps, they are just counting the original number of RNA molecules in the sample. So in this way, the relationship of RIN to qPCR is not the same as RIN to NanoString nCounter assays. Many NanoString applications also use FFPE samples for their RNA, and RIN is not considered a sensitive measure of RNA quality for FFPE samples.

That said, if there is a DV200 less than 50%, Nanostring mostly recommends increasing the sample input. It turns out that according to Nanostring there are MANY (the majority) of groups who do not even check the DV200, they just look at concentration, A260/A280 (1.7-2.3) and A260/A230 (1.8-2.3). They recommend including the DV200 metric if you think there is a reason your samples will be degraded (FFPE). There are many positive and negative controls within in the assay, as well as QC measurements of the data’s signal and quality after running the assay. If there are a lot of QC flags, then it’s possible that something is off and it is recommended to go back and run the sample on a bioanalyzer to double-check the DV200. Otherwise, the assay worked as expected. If the DV200 was poor, we would have expected to get poor signal/background ratios that raise a QC flag.

5) The authors indicate that PBS was used to “preserve host material”; is there a reference for this? As considerable or at least some lysis presumably happens before this step (due to vortexing), any lysed pathogenic/microbial nucleic acid material is preserved.

**AU:** Correct, during vortexing some mechanical lysis can happen and microbial material will carry over. Of course, that is why we run housekeeping genes to ensure host material was present. Our use of PBS to aid in removal of RNAlater was informed by methods presented in a manuscript entitled, “Variation of glucoraphanin metabolism in vivo and ex vivo by human gut bacteria.” Fei Li et al. PMID: 21342607 PMCID: PMC3137642 DOI: 10.1017/S0007114511000274. This reference has been added to the manuscript.

6) Line 193: at what temperature were the samples stored?

**AU:** -80°C. This has been added to the text.
